# MiR-101-containing extracellular vesicles bind to BRD4 and enhance proliferation and migration of trophoblasts in preeclampsia

**DOI:** 10.1186/s13287-020-01720-9

**Published:** 2020-06-11

**Authors:** Jinhui Cui, Xinjuan Chen, Shuo Lin, Ling Li, Jianhui Fan, Hongying Hou, Ping Li

**Affiliations:** 1 Department of Obstetrics and Gynecology, The Third Affiliated Hospital of Sun Yat-Sen University, No. 600, Tianhe Road, Guangzhou, 516000 Guangdong Province People’s Republic of China; 2 Department of Endocrinology, The Third Affiliated Hospital of Sun Yat-Sen University, Guangzhou, 516000 People’s Republic of China

**Keywords:** Preeclampsia, Extracellular vesicles, MicroRNA-101, Bromodomain-containing 4, Nuclear factor-kappa B, C-X-C motif chemokine ligand 11

## Abstract

**Background:**

Preeclampsia (PE) is a frequently occurring pregnancy disorder in the placenta, which results in various maternal and fetal complications. The current study aims to evaluate the role of extracellular vesicles (EVs)-encapsulated microRNA (miR)-101 in biological processes of trophoblasts in PE and its underlying mechanism.

**Methods:**

Human umbilical cord mesenchymal stem cell (HUCMSC) and HUCMSC-derived EVs were isolated and cultured, after which EV characterization was carried out using PKH67 staining. In silico analyses were adopted to predict the downstream target genes of miR-101, and dual luciferase reporter gene assay was applied to validate the binding affinity. Furthermore, loss- and gain-of-function approaches were adopted to determine the role of miR-101 and bromodomain-containing protein 4 (BRD4) in trophoblast proliferation and invasion using EDU staining and transwell assay. In addition, a rat model of PE was established to verify the function of EV-encapsulated miR-101 in vivo.

**Results:**

Placental tissues obtained from PE patients presented with downregulated miR-101 expression and upregulated BRD4 and CXCL11 expression. EV-encapsulated miR-101 from HUCMSCs could be delivered into the trophoblast HTR-8/SVneo cells, thus enhancing proliferation and migration of trophoblasts. Mechanically, miR-101 targeted and negatively regulated BRD4 expression. BRD4 knockdown promoted the proliferation and migration of trophoblasts by suppressing NF-κB/CXCL11 axis. EV-encapsulated miR-101 from HUCMSCs also reduced blood pressure and 24 h urine protein in vivo, thereby ameliorating PE.

**Conclusion:**

In summary, EV-encapsulated miR-101 promoted proliferation and migration of placental trophoblasts through the inhibition of BRD4 expression via NF-κB/CXCL11 inactivation.

## Background

Preeclampsia (PE), a common placenta-induced disorder during pregnancy, presents with clinical findings of hypertension and proteinuria [1, 2]. The development of PE has been associated with several underlying pathologies, including poor placentation, excessive maternal inflammation, and endothelial dysfunction [3]. Recently, fetal trophoblast invasion of maternal decidual vessels has been proposed to be indispensable during normal pregnancy [4]. Moreover, reduced trophoblast migration and invasion have been previously linked with the exacerbation of PE [5]. Therefore, elucidating the underlying mechanism by which placental trophoblast proliferation and invasion affect PE is crucial in order to attenuate PE.

Extracellular vesicles (EVs) consisting of microvesicles, apoptotic bodies, and syncytial nuclear aggregates play favorable roles in successful fetal development [6]. The pathogenesis of PE is also involved in the alteration in protein expression in the human umbilical cord mesenchymal stem cell (HUCMSC)-derived EVs [7]. A prior study noted that HUCMSCs-derived EVs could protect the morphology and angiogenesis of placenta in rats with PE [8]. MicroRNAs (miRs) are small and endogenous noncoding RNAs that regulate various biological processes including disease progression, cell proliferation, and PE prognosis and diagnosis [9]. Interestingly, miR-101 could regulate the apoptosis of trophoblast HTR-8/SVneo cells during PE [10]. In silico analyses in our study identified bromodomain-containing protein 4 (BRD4) to be one of the downstream targets of miR-101. BRD4 belongs to bromodomain and extra-terminal family and has a complex binding mechanism with acetylated lysine residues on histone tails [11]. The function of BRD4 has also been reported in human placentas obtained from patients with early PE [12]. Moreover, BRD4 could bind to acetylated lysine-310, thereby modulating the nuclear factor-κB (NF-κB) transcriptional activity [13]. As a transcription factor, NF-κB could modulate genes related to inflammation and immune response [14]. In addition, the inactivation of NF-κB signaling pathway was found to be one of the underlying factors involved in attenuated placental cell injury in PE mice [15]. Furthermore, the inactivation of NF-κB reportedly inhibits C-X-C motif chemokine ligand 11 (CXCL11) expression in retinal pigment epithelial cells [16] and CXCL11 is also associated with microbial stimuli to intestinal inflammation [17]. More importantly, altered expression of CXCL11 has been found in PE [18]. These findings unveil a possible mechanism underlying the involvement of EV-encapsulated miR-101 and BRD4-dependent NF-κB-CXCL11 axis in PE. Thus, in the current study, PE rat models and human extravillous trophoblast cell lines HTR-8/SVneo were established to explore the potential regulatory network. Understanding the molecular regulation of EV-encapsulated miR-101 is essential to develop therapeutic methods that can attenuate PE.

## Material and methods

### Ethical approval

This study was approved by the ethics committee of The Third Affiliated Hospital of Sun Yat-Sen University and was conducted in accordance with the *Declaration of Principles of Helsinki*. Ethical agreements were obtained from the donors or their relatives by written informed consent. The animal experiments were carried out in strict accordance with the recommendations provided by the Guide for the Care and Use of Laboratory Animals of the National Institutes of Health and was approved by the Animal Care and Use Committee of The Third Affiliated Hospital of Sun Yat-Sen University. Maximum efforts were made to minimize unnecessary distress in the animals.

### In silico analyses

The downstream regulatory genes of miR-101 were predicted by TargetScan (http://www.targetscan.org/vert_72/), RNAInter (http://www.rna-society.org/rnainter/), and StarBase (http://starbase.sysu.edu.cn/) and intersected with the eclampsia-related genes identified in the GeneCards database (https://www.genecards.org/) using Jvenn tool (http://jvenn.toulouse.inra.fr/app/example.html). The candidate genes were further screened with the use of Gene Ontology (GO) analysis through the online website KOBAS (http://kobas.cbi.pku.edu.cn/kobas3). To predict the downstream regulatory factors of genes, the Multi-Experiment Matrix (MEM) tool (https://biit.cs.ut.ee/mem/index.cgi) was used for co-expressed genes [19]. The threshold value was increased to find the eclampsia-related genes in the GeneCards database again, and the intersection of the co-expressed genes and eclampsia-related genes was obtained with the application of the Jvenn tool. The Coexpedia tool (http://www.coexpedia.org/) was used to search for interactions between co-expressed gene candidates, and finally, genes were selected based on the correlation score.

### Isolation and culture of HUCMSCs

Placental tissues were obtained from 30 PE female patients within the age of 20–36 years who underwent cesarean delivery and additional 30 placental tissues were collected from healthy donors. Tissues were collected immediately after delivery, washed with sterile phosphate-buffered saline (PBS), and quickly frozen in liquid nitrogen in the laboratory. A primary culture of HUCMSCs was established using a standard procedure. Briefly, umbilical cords were washed using 75% ethanol and subsequently washed further with Dulbecco’s modified Eagle’s medium (DMEM; Thermo Fisher Scientific, Waltham, MA, USA) supplemented with 1% L-glutamine, 10% fetal bovine serum (FBS; Thermo Fisher Scientific, USA), 100 μg/mL streptomycin, and penicillin solution. Following the removal of excess blood, the umbilical cords were sliced into small pieces (3–5 mm) and then incubated at 37 °C with 5% CO_2_ with the previous washed medium. Upon 80–90% HUCMSC confluence, cells were trypsinized and prepared for passage. Thereafter, HUCMSCs passaged less than 6 times were used in subsequent experiments. The morphological appearance of HUCMSCs was observed under a light microscopy (Olympus, Tokyo, Japan).

### Flow cytometry

HUCMSCs were trypsinized (2–4 min), washed with PBS in the absence of magnesium and calcium, and blocked with 10% normal goat serum to prevent nonspecific binding. Cells were probed with monoclonal primary antibodies with fluorescein isothiocyanate (FITC) staining for cluster of differentiation (CD) markers (CD14, CD19, CD29, CD34, CD44, CD45, CD73, CD90, CD105, and HLA-DR (1:100; BioLegend, San Diego, CA, USA)) for 30 min. Cells were resuspended in 10% normal goat serum and finally analyzed with a CyAn ADP Analyzer (Beckman Coulter, Brea, CA, USA).

### Induction of HUCMSC differentiation

Cells were cultured in osteogenic, adipogenic, or chondrogenic differentiation medium, respectively [osteogenesis differentiation kit, adipogenic differentiation kit, chondrogenic differentiation kit (Gibco, Carlsbad, CA, USA)], in 6-well plates for 2–3 weeks for HUCMSC differentiation. During this time, the medium was changed every 2–3 days. Cells were fixed with 4% formaldehyde for 30 min and stained with Alizarin Red S solution (pH 4.2) for 10 min. Osteogenic differentiation was observed under a microscopy (Nikon, Tokyo, Japan). Adipogenic differentiation was dyed using Oil Red O staining (Sigma-Aldrich, St Louis, MO, USA). Cartilage differentiation was determined using Alcian Blue staining.

### Purification and characterization of EVs

FBS were ultracentrifugated and centrifugated at 100,000×*g* for 18 h to remove EVs from the serum. HUCMSCs were cultured in a medium supplemented with 10% EV-free FBS (SBI, System Biosciences, Mountain View, CA, USA) for 72 h, followed by centrifugation at 1200×*g* for 25 min at 4 °C to remove the inside cell debris and dead cells, and then filtered through a 0.2-mm filter. EVs were resuspended in PBS.

Immunoblotting was adopted to determine the expression of EV-specific markers (HSP70, CD63, CD9, and GM130). The particle size distribution of EVs was analyzed by Nanoparticle Tracking Analysis (NTA; Malvern Instruments, Malvern, UK). Moreover, the morphology of EVs was observed using a transmission electron microscopy (TEM; Tecnai Spirit; FEI, USA).

### Reverse transcription quantitative polymerase chain reaction (RT-qPCR)

Total RNA from tissues and cells were isolated using TRIzol (Solarbio, Beijing, China). The concentration of RNA was measured and reversely transcribed into cDNA using one-step miR reverse transcription kit (D1801, HaiGeen, Harbin, China) and cDNA reverse transcription kit (K1622, Beijing Yaanda Biotechnology Co., Ltd., Beijing, China). Human-derived primers were synthesized (Table 1) by Takara (Dalian, China). Real-time PCR kit (ViiA7, Daan Gene, UK) was performed for real-time PCR. U6 and glyceraldehyde-3-phosphate dehydrogenase (GAPDH) were adopted as internal reference. The relative quantification method (2^-ΔΔCt^ method) was applied to calculate the relative transcription level of the target gene [20]. MiR-101 was detected in mice using miScript II RT kit and miScript SYBR Green PCR kit with a miScript Primer Assay kit (Qiagen, Hilden, Germany) in strict accordance with the manufacturer’s instructions. The primers included universal primers and miR-101-specific primers: SNORD61 (Hs_SNORD61_11; Cat # MS00033705; Qiagen) and Rn_miR-101a-3p (Cat # MS00012950; Qiagen). The expression level was calculated using the 2^-ΔΔCt^ method.

**Table 1:** Primer sequences for RT-qPCR

Name	Primer sequences (5′-3′)
miR-101	TACAGTACTGTGATAACTGAA
U6 (F)	CTCGCTTCGGCAGCACA
U6 (R)	AACGCTTCACGAATTTGCGT
BRD4 (F)	GAGCTACCCACAGAAGAAACC
BRD4 (R)	GAGTCGATGCTTGAGTTGTGTT
GAPDH (F)	GGAGCGAGATCCCTCCAAAAT
GAPDH (R)	GGCTGTTGTCATACTTCTCATGG

Notes: *RT-qPCR* reverse transcription quantitative polymerase chain reaction, *miR-101* microRNA-101, *BRD4* bromodomain-containing 4, *GAPDH* glyceraldehyde-3-phosphate dehydrogenase, *F* forward, *R* reverse

### Immunoblotting

Total protein was separated using sodium dodecyl sulfate-polyacrylamide gel electrophoresis, and 50 μg of protein was loaded for each sample. Afterwards, proteins in the gel were transferred onto a nitrocellulose membrane and then blocked with 5% skimmed milk solution dissolved in t-butyldimethylsilyl (TBS) solution. Next, the membrane was probed with specific human primary antibody while undergoing incubation at 4 °C overnight. After being washed 3 times (10 min/time) with Tris-buffered saline Tween (TBST), the membrane was re-probed with secondary antibody by incubation at room temperature for 1–2 h. A chemiluminescence system (Thermo, Euroclone, Milan, Italy) was adopted to analyze the relative gray value. The specific primary antibodies used are as follows: collagen type II alpha 1 chain (COL2A1; clone M2139; Santa Cruz), HSP70 (Abcam, Cambridge, UK), CD9 (Abcam, UK), anti-CD63 (Abcam, UK), anti-tumor susceptibility 101 (TSG101; Santa Cruz, CA, USA), anti-Golgi matrix protein 130 kD (GM130; Cell Signaling, Beverly, MA, USA), anti-BRD4 (Abcam, UK), anti-NF-κB (Abcam, UK), anti-CXCL11 (human; Abcam, UK), anti-CXCL11 (Rat; R & D systems, Minneapolis, MN, USA), anti-IL-6 (Abcam, UK), anti-TNF-α (Abcam), anti-p65 (Cell Signaling, USA), anti-p-IkBα (Abcam, UK), and anti-IkBα (Abcam, UK).

### Preparation and infection of lentiviral vectors

Lentiviral vectors containing miR-101 and its negative control and a plasmid containing wild-type (WT) or mutated (MUT) 3′-UTR BRD4 were designed and purchased from Genechem (Shanghai, China). Next, human extravillous trophoblast cell lines HTR-8/SVneo [(ATCC; American Type Culture Collection (Manassas, VA, USA)] were infected with these lentiviruses with a multiplicity of infection (MOI) of 20. Subsequently, cells were screened in medium with 1 μg/mL puromycin for 3 days. MiR-101 mimic labeled with cy3 (cy3- miR-101 mimic), miR-101 mimic, miR-101 mimic NC, short interfering RNA (siRNA) target BRD4, and BRD4 overexpression (oe-BRD4) plasmid vectors were designed and purchased from GenePharma (Shanghai, China). Lipofectamine 2000 (Invitrogen, Carlsbad, CA, USA) was used for infection according to the manufacturer’s instructions. GW4869 (10 μM; Sigma, California, USA) was used to inhibit the release of EVs.

### Identification of EVs through PKH67 labeling

PKH67 green fluorescent cell linker kit (Sigma-Aldrich, USA) was used for lipid bilayer labeling of EVs. According to the manufacturer’s instructions, EVs were first suspended in 100 mL of Diluent C and then added with 0.4 mL of PKH67 ethanol prepared into dye solution (4 × 10^6^ M). Then, 100 mL of the EV suspension was mixed with 100 mL of the dye solution. The cells and the dye suspension were incubated for 1 to 5 min. After periodic mixing, 200 mL of serum was added, incubated for 1 min to stop staining, and resuspended in a fresh sterile conical polypropylene tube. The mixed solution was centrifuged at 100,000*g* (4 °C) for 2 h. EVs were collected after being enriched at the density range of 1.13–1.19 g/mL.

### Dual luciferase reporter gene assay

Dual luciferase reporter gene assay was adopted to verify the target relationship between miR-101 and BRD4. HEK-293 T cells (ATCC) were co-transfected with miR-101 mimic or miR-NC and 3′-UTR luciferase vector (150 ng) [Lipofectamine 2000 (Invitrogen, USA)]. After being transfected for 24 h, cells were lysed and their luciferase activity was measured using a dual luciferase reporting kit (Beyotime, Shanghai, China) according to the manufacturer’s instructions. Subsequently, HEK-293 T cells were transfected with the BRD4 reporter gene vector to determine the effect of EV-encapsulated miR-101 (cancer-associated fibroblast culture medium) on the luciferase activity of BRD4 reporter gene. After 8 h of transfection, HEK-293 T cells were co-cultured with the extracted EVs for 24 h and were collected for lysis to measure their luciferase activity.

### 5-ethynyl-2′-deoxyuridine (EdU) staining

The cells were incubated with 50 mM Edu (RiboBio, Guangzhou, China) for 12 h and then fixed with 4% paraformaldehyde at 25 °C for 30 min. Next, the cells were incubated in PBS supplemented with 0.3% triton x-100 on a decoloring shaker for 10 min. The cells underwent further incubation in Apollo staining solution (RiboBio, China) for 20 min, then in NaCl/Pi (3 times, 10 min for each, room temperature), and finally with 4′-6-diamidino-2-phenylindole (DAPI, 1: 2500; Roche, Mannheim, Germany) at room temperature for 10 min.

### Transwell assay

Cell invasion ability was detected using the 24-well transwell system (Merck Millipor, Billerica, MA, USA) and Matrigel-pre-coated filter (BD, Bioscience Pharmingen, San Jose, CA, USA). Besides, the transwell migration test was performed using a transwell chamber (pore 0.8 μm; Merck Millipore, Billerica, MA, USA). The 50,000 cells were suspended in 200 μL of serum-free medium for each well and placed in the upper chamber. Meanwhile, the lower chamber was added with 600 μL of medium with 10% FBS as a chemotactic attractant. The transwell membrane was pre-coated with 50 μL of Matrigel (Corning, Midland, MI, USA) and then dried at 37 °C for 2 h. After 24 h of incubation, cells were fixed with 4% paraformaldehyde and stained with crystal violet. The filter was wiped using a small cotton swab to remove cells from the upper surface of the filter. Cells in 5 random non-overlapping regions were counted.

### Establishment of rat model of PE

Female Sprague-Dawley rats with the age of 10 weeks (200–250 g) and male rats (250–300 g) were obtained from experimental animal center of The Secondary Military Medical University (Shanghai, China) and were fed for 1 week, allowing them to adapt to the environment. One male rat and two female rats were cohabited in each cage, with a constant temperature from 16:00 to 8:00. The following day, the cohabited rats were separated and the vaginal plug was observed in the female rat and vaginal smear was prepared to detect the presence of sperm and epithelial cells. The presence of sperms was regarded as the first day of pregnancy.

Pregnant rats were numbered (1, 2, 3…) and injected in the abdomen with NG-nitro-L-arginine methyl ester (L-NAME) on the 7th to the 19th day (250 mg/kg/day) to induce PE. The sham-operated rats were injected with sterile normal saline in the same manner, whereas healthy rats underwent no treatment (over 10 rats/group). A BP-2010 automatic noninvasive blood pressure monitor (Softron, Beijing, China) and an automatic biochemical analyzer (Roche, Basel, Switzerland) were adopted to monitor the blood pressure and 24 h of urinary protein, respectively, on the 7th (before injection of L-NAME), 9th, and 13th day of pregnancy. If the blood pressure was over 30 mmHg and 24 h urinary protein was markedly increased, it was indicative of successful establishment of PE rat models.

### Treatments of rat models

The sham-operated rats and the healthy rats underwent the same treatments while rat model establishment was continued. PE modeled rats were randomly divided into 3 groups for further treatments (6 rats/group): PE modeled rats received intra-abdominal injection of 140 μg/mL EVs derived from HUCMSCs infected with lentiviral vector containing miR-101 since the 14th day of pregnancy for 6 days. Blood pressure and 24 h of urinary protein were monitored and recorded on the 15th, 17th, and 19th day of pregnancy.

After the rats were anesthetized using 3% sodium pentobarbital solution (Propbs, Beijing, China), the fetus and placenta were immediately removed by cesarean section. The fetus and placenta of normal size were counted. Subsequently, the umbilical cord connected to the fetus was cut off after removing the fetal membrane and umbilical cord. Finally, the fetus and placenta were respectively weighed on an analytical balance.

### Morphology of placental tissue

Placental tissues were cut to blocks (1 cm × 1 cm × 1 cm) and rinsed using sterile normal saline to remove blood and mucus on the surface. Tissues were fixed in 4% paraformaldehyde for 24 h, paraffin-embedded, and sliced to serial sections (3–4 μm), followed by hematoxylin-eosin (HE) staining, terminal deoxynucleotidyl transferase-mediated dUTP nick-end labeling (TUNEL) staining (at least 3 slices for each rat). Subsequently, slices were depafaffinized, debenzolized, and conventionally stained with HE. Finally, the placenta slices were photographed and the pathological changes were observed under a light microscope.

### TUNEL staining

The placenta slices were subjected to TUNEL staining (Roche, Basel, Switzerland). Then, the slices were observed under a microscope. Five positive fields were photographed and analyzed using ImageJ software. The cell apoptosis rate was calculated as TUNEL positive cell number/total cell numbers × 100%.

### Statistical analysis

SPSS 21.0 (IBM Corp, Armonk, NY, USA) was applied for data analysis. The measurement data were presented as mean ± standard deviation of 3 independent tests. The comparison of normally distributed data between two groups was analyzed using *t* test. Comparisons among multiple groups were conducted using one-way analysis of variance (ANOVA). The correlation between miR-101 and BRD4 is obtained by Pearson’s correlation analysis. *p* < 0.05 was considered as statistically significant.

## Results

### HUCMSC-derived EVs encapsulate miR-101

**Fig. 1:**
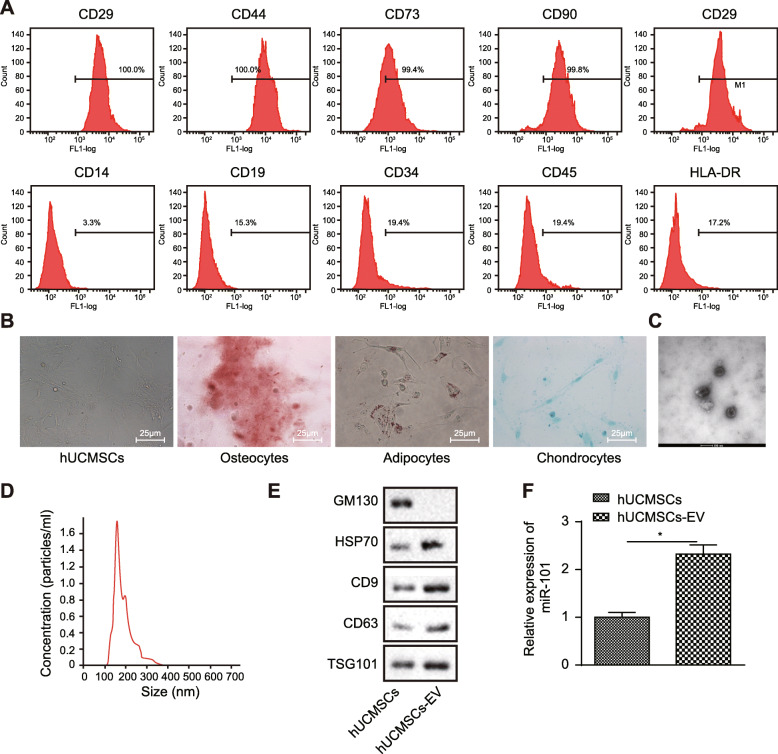
HUCMSC-secreted EVs encapsulate miR-101. **a** Expression of CD14, CD19, CD29, CD34, CD44, CD45, CD73, CD90, and HLA-DR determined by flow cytometry. **b** The morphology of HUCMSCs (bar = 50 μm) observed in inverted microscope. Osteogenic, adipogenic, and cartilage differentiation was dyed with Alizarin Red S staining, Oil Red O staining, and Alcian Blue staining, respectively. **c** The morphology of HUCMSC-derived EV determined by TEM (100 nm). **d** Size distribution of the HUCMSC-exosome analyzed by NTA. **e** The protein levels of GM130, CD63, and TSG101 determined by immunoblotting. **f** The expression of miR-101 in HUCMSCs and HUCMSC-EV relative to U6 determined by RT-qPCR. *p < 0.05 vs. **p < 0.01

Flow cytometry (Fig. 1a) was used to detect the expression of surface markers of HUCMSCs, demonstrating that HUCMSC surface markers CD29 (100.0%), CD44 (100.0%), CD73 (99.4%), CD90 (99.8%), and CD105 (100.0%) exhibited positive expression, whereas, CD14 (3.3%), CD19 (1.3%), CD34 (2.4%), CD45 (3.4%), and HLA-DR (1.2%) showed negative expression, conforming to the biological characteristics of HUCMSCs. Pictures of cell morphology were obtained as shown in Fig. 1b. Osteogenic, adipogenic, or chondrogenic differentiation experiments were carried out to further explore the differentiation capability of HUCMSCs, and results displayed that cells exhibited potential for osteogenesis, differentiation into fat or cartilage, indicating the successful isolation of HUCMSCs.

A prior study noted that miR-101 regulates apoptosis of trophoblast HTR-8/SVneo cells during PE [10], which led to us exploring the function of EV-encapsulated miR-101 during the early phase of PE. We isolated EVs from HUCMSCs and then characterized these EVs. Firstly, the TEM image revealed that the particle was uniformly circular or oval-shaped membranous vesicles with a diameter ranging from 40 to 150 nm. The membrane structure could be seen on the periphery of the vesicle with a low electron density component in the center (Fig. 1c). NTA results displayed that the diameter was approximately 61.58 nm (Fig. 1d). The expression of CD9, CD63, HSP70, and TSG101 on the EVs as well as cytoplasmic protein marker GM130 was determined with the application of immunoblotting, the results of which showed a significantly higher content of surface markers CD9, CD63, HSP70, and TSG101 in EVs compared with that in HUCMSCs, with the absence of GM130, suggesting the successful isolation of EVs (Fig. 1e). Subsequently, RT-qPCR was conducted for the assessment of miR-101 expression in HUCMSCs and their secreted EVs. As predicted, results showed that miR-101 expression in HUCMSC-EV was higher than its expression in HUCMSCs (Fig. 1f), indicating that miR-101 was encapsulated in EVs secreted by HUCMSCs.

### MiR-101 from HUCMSCs can be delivered into the HTR-8/SVneo cells via EVs

**Fig. 2:**
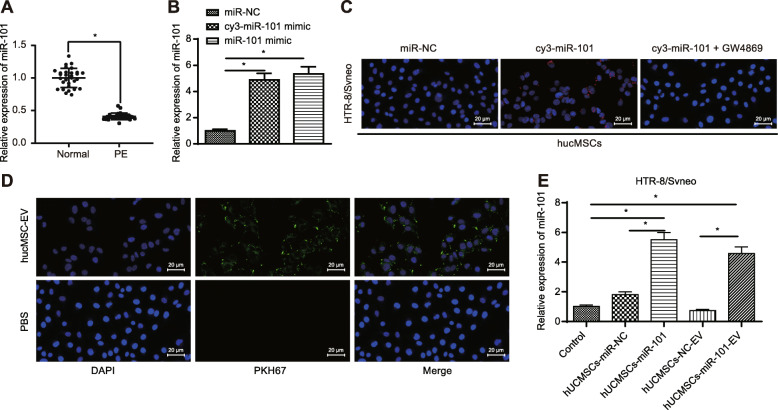
EV-encapsulated miR-101 from HUCMSCs can be delivered into the HTR-8/SVneo cells. **a** Expression of miR-101 in placental tissues in patients with PE compared to healthy donors measured by RT-qPCR. **b** Transfection efficiency of HUCMSCs transfected with cy3-miR-101 mimic, miR-101 mimic, or miR-NC determined using RT-qPCR. **c** Fluorescence result of HTR-8/SVneo cells after being co-cultured with HUCMSCs transfected with miR-NC, cy3-miR-101, or cy3-miR-101 + GW4869 observed under a fluorescence microscope (scale bar = 20 μm). **d** Fluorescence result of HTR-8/SVneo cells after being co-cultured with EVs which were labeled with PKH67 and secreted from HUCMSCs transfected with cy3-miR-101 mimic, observed under a fluorescence microscope (scale bar = 20 μm). **e** Expression of miR-101 in HTR-8/SVneo cells after being co-cultured with HUCMSC-miR-NC, HUCMSC-miR-101, HUCMSC-NC-EV, and HUCMSC-miR-101-EV determined by RT-qPCR. *p < 0.05 vs. **p < 0.01

A prior study reported that HUCMSC-derived EVs protect the morphology and angiogenesis of the placenta in rats with PE [8], and the regulatory role of miR-101 has been identified to play a functional role on the apoptosis of trophoblast HTR-8/SVneo cells during PE [10]. Thus, we hypothesized that miR-101 from HUCMSCs could be delivered into the placental trophoblasts via EVs, thereby exerting its influence on PE. To test this hypothesis, we adopted RT-qPCR (Fig. 2a) to measure the expression of miR-101, which showed suppressed miR-101 expression in placental tissues in PE patients compared to the placental tissues in healthy donors. Moreover, we successfully transfected HUCMSCs with miR-101 mimic labeled with cy3 (cy3-miR-101 mimic, the transfection efficiency was shown in Fig. 2b). While co-culturing HUCMSCs and HTR-8/SVneo cells, red fluorescence from cy3-miR-101 mimic was observed in HTR-8/SVneo cells via fluorescence microscope. However, when GW4869 was added to inhibit EVs released from HUCMSCs, co-culturing of transfected HUCMSCs and HTR-8/SVneo cells, no red fluorescence was observed from cy3-miR-101 mimic in HTR-8/SVneo cells (Fig. 2c). Together, these results suggested that miR-101 from HUCMSCs could be delivered into HTR-8/SVneo cells and this transportation may rely on EVs.

To further validate this hypothesis, we extracted the EVs secreted by HUCMSCs after transfection with cy3-miR-101 mimic and co-cultured with HTR-8/SVneo cells after labeling their lipid bilayer with PKH67, which displayed green fluorescence. Subsequently, both red and green fluorescence in HTR-8/SVneo cells were observed (Fig. 2d), indicating that miR-101 from HUCMSCs could be delivered into HTR-8/SVneo cells via EVs. We also co-cultured HTR-8/SVneo cells with HUCMSC cells transfected with miR-NC or miR-101 as well as co-cultured HTR-8/SVneo cells with HUCMSC-NC-EV and HUCMSC-miR-101-EV, respectively. RT-qPCR (Fig. 2e) results revealed a markedly increased expression of miR-101 in HTR-8/SVneo cells while co-culturing with either HUCMSC cells transfected with miR-101 or miR-101 transfected HUCMSC cells secreted EVs, compared with their corresponding NCs.

### EV-encapsulated miR-101 from HUCMSCs downregulates BRD4 expression in the HTR-8/SVneo cells

**Fig. 3:**
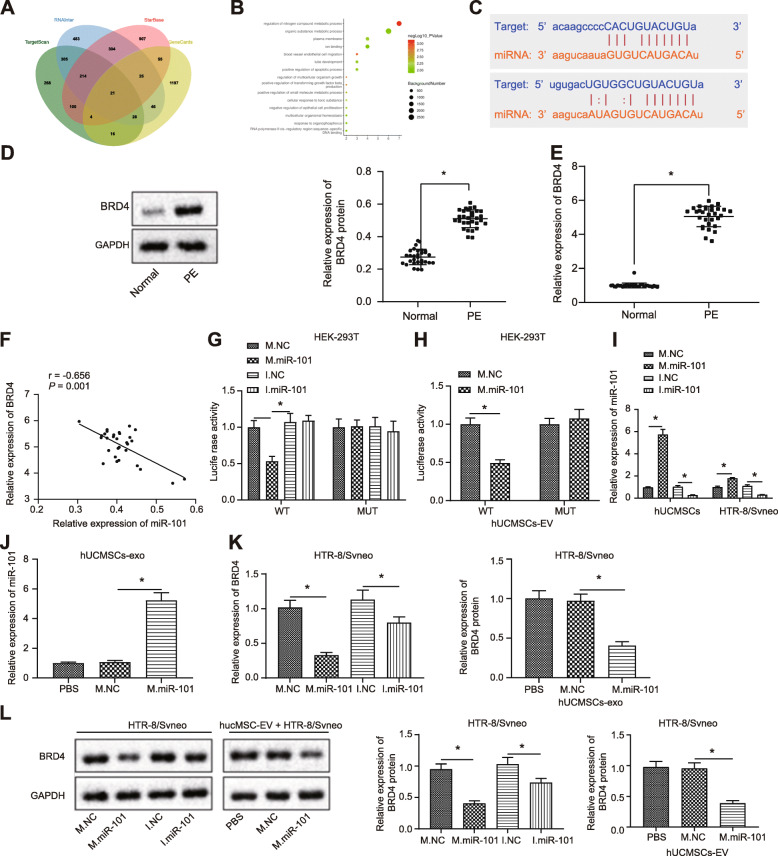
EV-encapsulated miR-101 from HUCMSCs downregulates BRD4 expression in the HTR-8/SVneo cells. **a** Intersection of downstream regulatory genes of miR-101 predicted by TargetScan, RNAInter, and StarBase with genes related to eclampsia in the GeneCards database. **b** Enrichment result of candidate genes by GO analysis from the online KOBAS. The *x*-axis indicates the number of genes involved, while the *y*-axis indicates the GO entry name. The dot color indicates −log10 *p* value, and the dot size indicates the GO entry background gene number. **c** Binding relationship between miR-101 and BRD4 predicted by bioinformatic analysis. **d**, **e** mRNA and protein expression of BRD4 in the placental tissues in PE patients and healthy donors determined by RT-qPCR and immunoblotting. **f** Correlation between miR-101 and BRD4 in PE determined by Pearson’s correlation analysis. **g** The targeting relationship between miR-101 and BRD4 determined by dual luciferase reporter gene assay. **h** The targeting relationship between EV-encapsulated miR-101 and BRD4 determined by dual luciferase reporter gene assay. **i** The transfection efficiency of miR-101 in HTR-8/SVneo cells and in EVs isolated from HUCMSCs infected with miR-101 mimic. **j** The transfection efficiency of EV-encapsulated miR-101 in HTR-8/SVneo cells and in EVs isolated from HUCMSCs infected with miR-101 mimic. **k**, **l** The mRNA and protein expression of BRD4 after miR-101 or EV-encapsulated miR-101 treatment. *p < 0.05 vs. **p < 0.01. WT, wild-type 3′-UTR BRD4; MUT, mutated type 3′-UTR BRD4; M.NC, negative control mimic; M.miR-101, microRNA-101 mimic; I.NC, negative control inhibitor; I.miR-101, miR-101 inhibitor

To better elucidate the role of miR-101 in PE, the bioinformatic websites TargetScan, RNAInter, and StarBase Gene predicted 956, 1426, and 1630 downstream regulatory genes of miR-101, respectively. Besides, 1392 genes were found to be related to eclampsia. A total of 21 genes were intersected among these screened genes (Fig. 3a). In order to further screen genes, GO analysis was performed on candidate genes through the online website KOBAS (Fig. 3b). Results revealed that most genes were involved in the regulation of nitrogen compound metabolic process with the smallest *p* value. Regarding the genes involved in biological process, combining prior findings suggesting that BRD4 was involved in human placentas from patients with early PE [12], BRD4 was selected as the gene of interest for subsequent studies. In addition, the binding relationship between miR-101 and BRD4 was predicted by bioinformatic analysis (Fig. 3c).

Moreover, we compared the expression of BRD4 in the placental tissues of patients with PE and that of healthy donors. RT-qPCR and immunoblotting results (Fig. 3d, e) showed that the protein and mRNA expression of BRD4 were higher in the placental tissues of patients with PE than those of placental tissues from healthy donors. In addition, Pearson’s correlation analysis revealed a negative correlation between miR-101 and BRD4 in PE as reflected in Fig. 3f. Dual luciferase reporter gene assay further verified that EV-encapsulated miR-101 secreted by HUCMSCs bound to 3′-UTR region of BRD4 (Fig. 3g, h). Then, miR-101 was successfully upregulated or downregulated in HTR-8/SVneo cells. HUCMSCs were transfected with miR-101 mimic, and EVs were isolated from HUCMSCs infected with miR-101 mimic (Fig. 3i, j). RT-qPCR and immunoblotting results (Fig. 3k, l) displayed that BRD4 expression in HTR-8/SVneo cells was reduced by both the direct overexpression of miR-101 and the addition of EV-encapsulated miR-101. Taken together, these findings suggest that EV-encapsulated miR-101 obtained from HUCMSCs decreased the expression of BRD4 in the HTR-8/SVneo cells.

### EV-encapsulated miR-101 enhances proliferation and migration of placental trophoblasts by repressing BRD4 expression

**Fig. 4:**
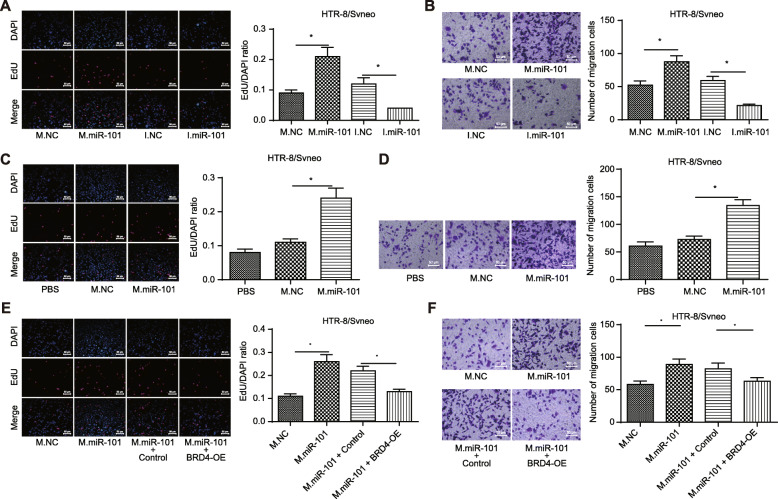
EV-encapsulated miR-101 from HUCMSCs promotes proliferation and migration of placental trophoblasts by inhibiting BRD4 expression. **a** Cell proliferation of HTR-8/SVneo cells in response to miR-101 mimic, miR-101 inhibitor, or their corresponding NCs determined by EDU staining (scale bar = 50 μm). **b** Cell migration of HTR-8/SVneo cells in response to transfection with miR-101 mimic, miR-101 inhibitor, or their corresponding NCs determined by transwell assay (scale bar = 50 μm). **c** Cell proliferation of HTR-8/SVneo cells in response to addition of EV released from HUCMSCs transfected with miR-101 mimic determined by EDU staining (scale bar = 50 μm). **d** Cell migration of HTR-8/SVneo cells in response to addition of EV released from HUCMSCs transfected with miR-101 mimic determined by transwell assay (scale bar = 50 μm). **e** Cell proliferation in HTR-8/SVneo cells transfected with miR-101 mimic and oe-BRD4 determined by EDU staining (scale bar = 50 μm). **f** Cell migration in HTR-8/SVneo cells transfected with miR-101 mimic and oe-BRD4 determined by transwell assay (scale bar = 50 μm). *p < 0.05 vs. **p < 0.01. M.NC, negative control mimic; M.miR-101, microRNA-101 mimic; I.NC, negative control inhibitor; I.miR-101, miRNA-101 inhibitor; BRD4-OE, overexpressed bromodomain-containing protein 4 plasmid

To better understand the function of miR-101-BRD4 axis in the proliferation and migration of placental trophoblasts, we upregulated or downregulated miR-101 in HTR-8/SVneo cells and also isolated EVs from HUCMSCs transfected with miR-101 mimic. EDU staining and transwell results (Fig. 4a–d) revealed that overexpression of miR-101 as well as EV-encapsulated miR-101 from HUCMSCs induced promoted proliferation and migration of HTR-8/SVneo cells compared to those transfected with mimic NC. Accordingly, an opposite trend was observed in response to miR-101 silencing. In addition, EDU staining and transwell results of HTR-8/SVneo cells transfected with miR-101 mimic and oe-BRD4 (Fig. 4e, f) revealed that oe-BRD4 treatment counteracted the promotion effect of miR-101 mimic on proliferation and migration of HTR-8/SVneo cells. Coherently, it was concluded that EV-encapsulated miR-101 from HUCMSCs promoted proliferation and migration of placental trophoblasts by repressing BRD4 expression.

### BRD4 repression enhances proliferation and migration of placental trophoblasts by repressing NF-κB/CXCL11 axis

**Fig. 5:**
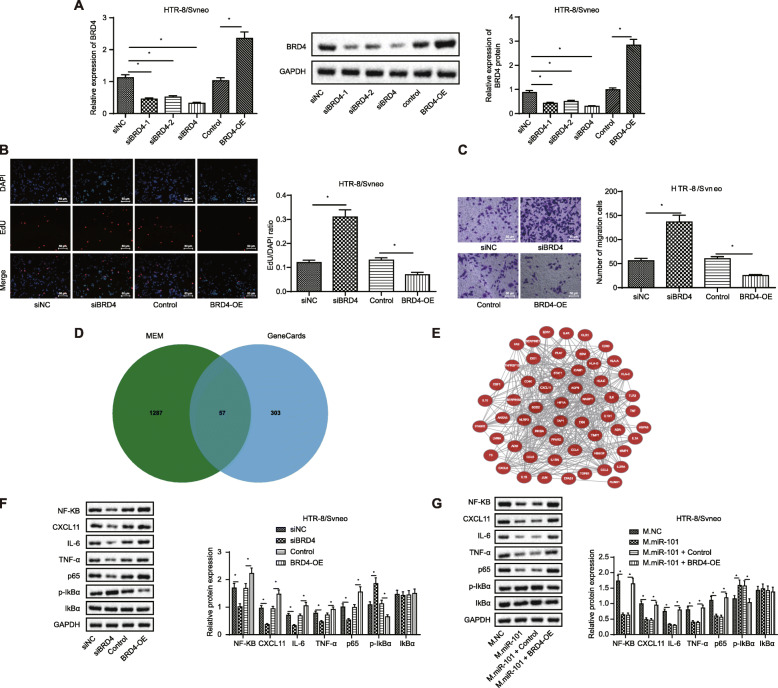
BRD4 downregulation promotes proliferation and migration of placental trophoblasts by inhibiting NF-κB/CXCL11 axis. BRD4 was overexpressed or repressed in HTR-8/SVneo cells. **a** Overexpression and silencing efficiency of BRD4 in HTR-8/SVneo cells determined by RT-qPCR and Western blot analysis. **b** Cell proliferation of HTR-8/SVneo cells after treatments determined by EDU staining (scale bar = 50 μm). **c** Cell migration of HTR-8/SVneo cells after treatments determined by transwell assay (scale bar = 50 μm). **d** Venn map of intersected genes between NF-KB-related co-expression genes retrieved from MEM and eclampsia-related genes in the GeneCards database. **e** Network diagram of co-expression relationships among 57 candidate genes in the Coexpedia. **f** Expression of NF-KB, CXCL11 IL-6, TNF-α, p65, p-IkBα, and IkBα in HTR-8/SVneo cells. **g** The expression of NF-KB, CXCL11 IL-6, TNF-α, p65, p-IkBα, and IkBα in HTR-8/SVneo cells in response to mimic NC, miR-101 mimic, miR-101 mimic + control, and miR-101 mimic + oe-BRD4. *p < 0.05 vs. **p < 0.01. M.NC, negative control mimic; M.miR-101, microRNA-101 mimic; I.NC, negative control inhibitor; I.miR-101, miRNA-101 inhibitor; si-BRD4, short interfering RNA target BRD4; si-NC, negative control of si-BRD4; BRD4-OE, overexpressed bromodomain-containing protein 4 plasmid

To better elucidate the role of BRD4 in the proliferation and migration of placental trophoblasts, thereby exerting its function on PE, we successfully overexpressed or repressed BRD4 expression in HTR-8/SVneo cells (Fig. 5a), and si-BRD4 was found with the highest silencing efficiency and was selected for subsequent experiments. EDU staining and transwell results (Fig. 5c, d) revealed that knocking down of BRD4 promoted proliferation and migration of HTR-8/SVneo cells compared to si-NC group, and accordingly, an opposite trend was observed in response to BRD4 overexpression.

A prior study has showed that BRD4 can bind to acetylated lysine-310, thereby modulating the NF-κB transcriptional activity [13], and the inactivation of NF-κB signaling pathway is concomitant with the attenuated injury of placenta cell in mice with PE [15]. Moreover, 57 genes were obtained by intersecting 2136 NF-KB-related co-expression genes identified by MEM with 360 genes related to eclampsia in the GeneCards database (Fig. 5d). Coexpedia was used to obtain a co-expression network among 57 candidate genes (Fig. 5e), and finally, 10 genes were selected based on their correlation score (Table 2). Among these 10 genes, the CXCL11 expression has been reported to be inhibited by the inactivation of NF-κB in retinal pigment epithelial cells [16]. In our study, immunoblotting results (Fig. 5f, g) revealed that knockdown of BRD4 resulted in the decreased expression of NF-KB, CXCL11, IL-6, TNF-α, and p65, but the increased phosphorylation of IkBα. Accordingly, the opposite trend was observed in response to the overexpression of BRD4. In addition, the overexpression of BRD4 treatment could potentially counteract the inhibitory effect of miR-101 mimic on these downstream genes in HTR-8/SVneo cells.

**Table 2:** Score of co-expression gene candidates from Coexpedia

Rank	Gene symbol	Score
1	HLA-C	1651.453
2	HLA-A	1320.77
3	HLA-G	1078.813
4	HLA-E	920.443
5	TAP1	446.92
6	B2M	440.47
7	STAT1	351.773
8	IL1B	235.617
9	CCL5	232.921
10	CXCL11	224.754

Notes: *HLA* major histocompatibility complex, *STAT1* signal transducer and activator of transcription 1, *IL1B* interleukin 1 beta, *CCL5* C-C motif chemokine ligand 5, *CXCL11* C-X-C motif chemokine ligand 11

### EV-encapsulated miR-101 ameliorates PE by repressing BRD4 expression and thus inhibiting NF-κB/CXCL11 axis in vivo

**Fig. 6:**
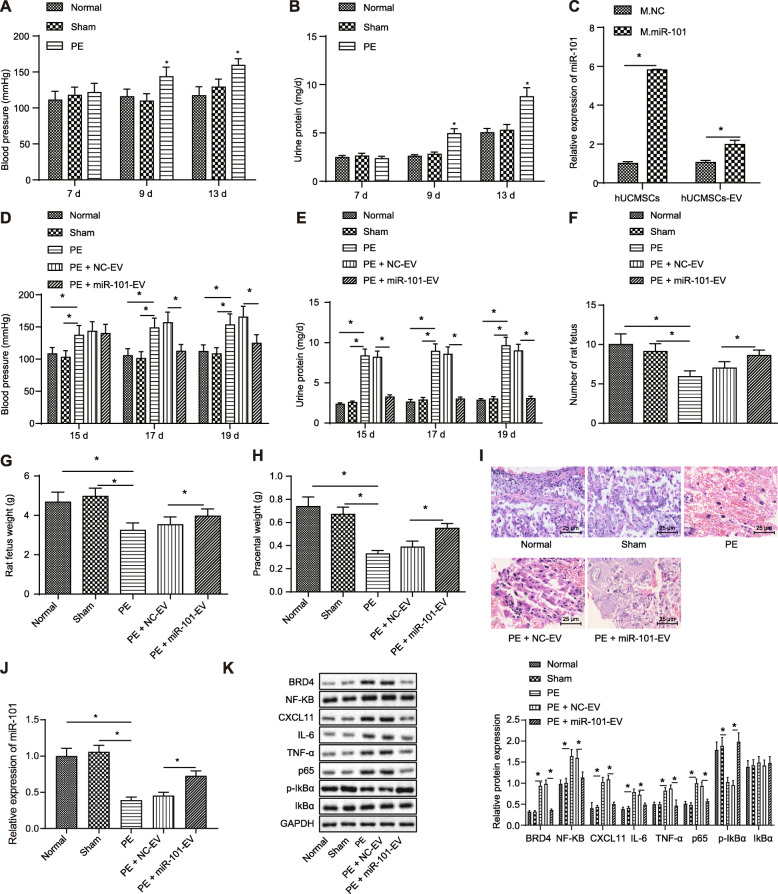
EV-encapsulated miR-101 attenuates PE by repressing BRD4 expression via inhibition of NF-κB/CXCL11 axis. **a** Blood pressure observed at the 15th, 17th, and 19th day in PE modeled rats, healthy rats and sham-operated rats. **b** 24 h urine protein observed at the 15th, 17th and 19th day in PE modeled rats, healthy rats and sham-operated rats. **c** Overexpression efficiency of miR-101 in HUCMSCs and HUCMSC-derived EVs determined using RT-qPCR. **d** Blood pressure observed at the 15th, 17th and 19th day in PE modeled rats, healthy rats, sham-operated rats, PE modeled rats treated with NC-EV, or miR-101-EV. **e** 24 h urine protein observed at the 15th, 17th, and 19th day in PE modeled rats, healthy rats, sham-operated rats, PE modeled rats treated with NC-EV, or miR-101-EV. **f**–**h** The weight of fetus and placenta in PE modeled rats, healthy rats, sham-operated rats, PE modeled rats treated with NC-EV, or miR-101-EV. **i** Placenta stained by HE staining in PE modeled rats, healthy rats, sham-operated rats, PE modeled rats treated with NC-EV, or miR-101-EV (scale bar = 25 μm). **j** Expression of miR-101 in PE modeled rats, healthy rats, sham-operated rats, PE modeled rats treated with NC-EV, or miR-101-EV determined by RT-qPCR. **k** Expression of BRD4, NF-KB, CXCL11, IL-6, TNF-α and p65 and IkBα in PE modeled rats, healthy rats, sham-operated rats, PE modeled rats treated with NC-EV, or miR-101-EV determined by immunoblotting. *p < 0.05

PE rat models were established to further establish the role of miR-101 in vivo, with their blood pressure (Fig. 6a) monitored, which demonstrated the blood pressure of PE modeled rats (158.91 ± 8.59 mmHg) was 30 mmHg higher than those of the healthy rats and sham-operated rats. Moreover, 24-h urinary protein (Fig. 6b) was determined respectively at the 9th and 13th day of their pregnancy, revealing that the urine protein of PE modeled rats was 4.92 ± 0.54 mg/d, gradually increased compared with those of healthy rats and sham-operated rats over time. Collectively, these results suggested that the PE rat models were successfully established.

Subsequently, we successfully injected PE modeled rats with EVs derived from HUCMSCs transfected with lentiviral vector containing oe-miR-101 or its NC (Fig. 6c). Blood pressure and 24-h urinary protein of these PE rats on the 15th, 17th, and 19th day of pregnancy were recorded (Fig. 6d, e). Results revealed that markedly lower blood pressure and 24-h urinary protein were observed at the 15th, 17th, and 19th day in PE modeled rats injected with miR-101-EV, compared to the NC-EV group. The fetus and placenta of normal size were weighed 19 days later (Fig. 6f–h), and results displayed that miR-101-EV treatment partially restored the adverse effects of fetus and placental weight during PE. Furthermore, fetus and placenta underwent HE staining (Fig. 6i), which demonstrated that PE rat models and those with NC-EV exhibited obvious infiltration of inflammatory cells, increased vacuole cells, and thickened basal area characterized by decidual swelling and thickening. However, miR-101-EV treatment attenuated the pathological changes of PE in these PE rats. RT-qPCR results (Fig. 6j) demonstrated that miR-101 expression was markedly upregulated in the placenta of the normal and sham-operated rats than that of PE modeled rats, and PE modeled rats injected with miR-101-EV also exhibited overexpressed expression of miR-101. Moreover, immunoblotting results (Fig. 6k) revealed that the overexpression of miR-101 resulted in the decreased expression of BRD4, NF-KB, CXCL11, IL-6, TNF-α, and p65, but the increased phosphorylation of IkBα. Therefore, EV-encapsulated miR-101 attenuated PE by suppressing BRD4 expression by inhibiting the NF-κB/CXCL11 axis.

## Discussion

**Fig. 7:**
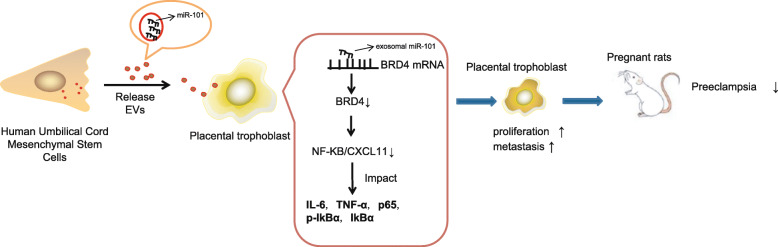
Schematic diagram of the proposed mechanism that EV-encapsulated miR-101 released from HUCMSCs promotes proliferation and migration of trophoblasts by downregulating BRD4 via inhibition of NF-κB/CXCL11, thereby attenuating PE

PE is a vascular disease that occurs during pregnancy and results in the impairment of placental development [21]. The differentiation of trophoblasts in the placenta could form the multinuclear layer-covering placenta, playing an essential role in the etiology of PE [22]. The current study was conducted with the main objective of determining the mechanism by which miR-101 encapsulated in EV released from HUCMSCs affects proliferation and migration of trophoblasts in vitro and the blood pressure and urine protein in a rat model of PE in vivo. Our key findings provide evidence that EV-encapsulated miR-101 promotes proliferation and migration of trophoblasts by downregulating BRD4 via NF-κB/CXCL11 suppression, thereby alleviating PE (Fig. 7).

Initially, we found that miR-101 encapsulated in HUCMSC-derived EVs could be delivered to trophoblast HTR-8/SVneo cells. The ability of HUCMSC-derived EVs to provide protection for the morphology and angiogenesis of the placenta in PE rats has been established in a previous study [8]. EV-encapsulated miRs derived from MSCs have been reported to serve as possible circulating biomarkers for the early detection of PE [23]. Based on findings obtained from prior miR profiling analysis and RT-qPCR, miR-101-3p has been identified to be downregulated in the plasma of unexplained recurrent spontaneous abortion [24], and miR-101-3p was also identified to be encapsulated in MSC-derived EVs during osteogenic differentiation [25]. Moreover, the inhibitory role of miR-101 in the migration and invasion of trophoblast cells has also been unveiled in PE [26]. Upon additional exploration, we found that the expression of miR-101 was downregulated in placental tissues in PE patients. EV-encapsulated miR-101 from HUCMSCs promoted the proliferation and migration of trophoblasts in vitro, thereby attenuating PE. In addition, lower levels of blood pressure and 24-h urine protein are indicative of less severe PE in the context of placental diseases [27]. Consistent with our in vivo results, EV-encapsulated 101 treatment reduced blood pressure and 24-h urinary protein.

Furthermore, in silico analyses conducted in our study identified BRD4 as one of the downstream targets by miR-101. We demonstrated that EV-encapsulated miR-101 targeted and negatively regulated BRD4, and thus this repression also promoted the proliferation and migration of trophoblasts*.* In human placentas obtained from PE patients, BRD4 expression was found to be upregulated, which contributes to the etiology of PE by regulating inflammation and anti-angiogenetic factors in human primary trophoblasts [12]. The inhibition of BRD is capable of reducing inflammation that occurs during myometrial contractions and rupture of fetal membranes, thereby lessening the risk of spontaneous preterm labor [28].

BRD4 has been documented to activate NF-κB pathway in gastrointestinal stromal tumor [29]. The inhibition of NF-κB is positively associated with the inhibition of vascular disease development, including PE [30]. Contrariwise, the activation of NF-κB pathway impairs the invasion of trophoblast, thus exacerbating PE [31]. Moreover, the activation of NF-κB can stimulate the release of CXCL11 in non-small cell lung cancer cells [32]. CXCL11 expression is markedly upregulated in patients with PE, serving as biomarker for PE [18]. Our findings suggested that BRD4 could inhibit the expression of NF-κB/CXCL11 axis, and the inhibition of NF-κB/CXCL11 axis promoted proliferation and migration of trophoblasts.

## Conclusion

In conclusion, our findings further highlighted the mechanism underlying the effect of EV-encapsulated miR-101 from HUCMSCs on PE. Particularly, EV-encapsulated miR-101 could downregulate BRD4 expression, thereby suppressing the expression of NF-κB/CXCL11 axis. The aforementioned mechanism could promote the proliferation and migration of trophoblasts in vitro, thereby reducing blood pressure and 24-h urinary protein in a rat model of PE in vivo, suggesting that EV-encapsulated miR-101 could serve as a therapeutic target for PE in the future. However, only a single dose of EVs was adopted in our experiments. Therefore, the optimum dose and the administration frequency should be explored in upcoming studies. Moreover, the regulatory role of BRD4 and CXCL11 in proliferation and migration of trophoblasts requires additional validation.

## Data Availability

Not applicable.

## Abbreviations

PE: Preeclampsia
EVs: Extracellular vesicles
miR: MicroRNA
HUCMSCs: Human umbilical cord mesenchymal stem cells
BRD4: Bromodomain-containing protein 4
NF-κB: Nuclear factor-κB
CXCL11: C-X-C motif chemokine ligand 11
MEM: Multi-Experiment Matrix
PBS: Phosphate buffered saline
FBS: Fetal bovine serum
FITC: Fluorescein isothiocyanate
CD: Cluster of differentiation
RT-qPCR: Reverse transcription quantitative polymerase chain reaction
GAPDH: Glyceraldehyde-3-phosphate dehydrogenase
TBS: t-Butyldimethylsilyl
TBST: Tris-buffered saline Tween
WT: Wild type
MUT: Mutated
oe-BRD4: BRD4 overexpression
EdU: 5-ethynyl-2′-deoxyuridine
L-NAME: NG-nitro-L-arginine methyl ester
